# Does implant material make a difference to the outcome of scoliosis surgery? Randomized controlled trial by cobalt-chrome rods and titanium rods

**DOI:** 10.1186/1748-7161-10-S1-P30

**Published:** 2015-01-19

**Authors:** Katsushi Takeshita, Masato Tanaka, Jun Takahashi, Daisuke Sakai

**Affiliations:** 1 The University of Tokyo, Japan; 2 Okayama University, Japan; 3 Shinshu University, Japan; 4 Tokai University, Japan

## Objective

The object of this study was to introduce the randomized controlled trial by cobalt-chrome rods and titanium rods in scoliosis surgery.

## Material and methods

Though cobalt-chrome and titanium-composite are two major metals used as a rod of spinal instrumentation, there is a paucity of superiority of one material over the other, especially with a high-level evidence.

## Results

Four authors of academic hospitals designed and are now performing on this trial supported by Stryker Japan after permission of Institutional Review Board of each hospital.

Candidates are female patients with an age between 10 to 19 with adolescent idiopathic scoliosis which are planned to have surgical treatment by segmental pedicle screw system. Curve profile should be Lenke type 1 to 3, over 45 degrees of the main curve by the Cobb method. We get written informed consent from patients and parents. Exclusion criteria are patients who cannot understand Japanese, patients who have had previous spinal surgery, patients who are pregnant, patient who have allergy to nickel, chrome, or titanium, etc.

Randomization to cobalt-chrome rod or titanium rod is performed with a web-based register adjusted by age, the Cobb angle of the main curve in standing, that in active bending, and the Risser grade. We set a total of 80 cases after power calculation by past reports.

Placement of pedicle screws and techniques of correction maneuver are to be reported. Radiographic parameters by X-ray, CT scan, and MRI at preoperative time, post operative time, and one-year after surgery are to be analyzed. Questionnaires of patient-reported outcome by SRS-22, SF-12, and SJ-27 (AIS-specifc outcome made by Japanese Scoliosis Society) are asked to fill at preoperative time, six-month after surgery, and one-year after surgery.

## Conclusion

We designed a practical randomized controlled trial of scoliosis surgery by two different-material rods. The study is now on going.

